# Role of Nongovernmental Organizations in Controlling COVID-19

**DOI:** 10.1017/dmp.2021.60

**Published:** 2021-03-04

**Authors:** Mohammad Mohseni, Saber Azami-Aghdash, Haleh Mousavi Isfahani, Ahmad Moosavi, Mozhgan Fardid

**Affiliations:** 1 Health Management and Economics Research Center, Iran University of Medical Sciences, Tehran, Iran; 2 Tabriz Health Services Management Research Center, Health Management and Safety Promotion Research Institute, Tabriz University of Medical Sciences, Tabriz, Iran; 3 School of Health Management and Information Sciences, Iran University of Medical Sciences, Tehran, Iran; 4 Department of Health and Community Medicine, Dezful University of Medical Sciences, Dezful, Iran; 5 Center for Health Related Social and Behavioral Sciences Research, Shahroud University of Medical Sciences, Shahroud, Iran

Coronavirus disease 2019 (COVID-19) is a contagious disease caused by a novel coronavirus. The virus spreads mainly through saliva droplets or nasal discharge when the infected person coughs or sneezes.^1^ Due to the high prevalence rate of COVID-19 and its associated problems, health-care systems are usually unable to manage and control it by themselves.^2^ Many health-care systems around the world have struggled to manage and control the outbreak without outside assistance. In response, numerous countries, such as Iran, have made efforts to use the capabilities and capacities of other sectors, such as the military, private sector, and public volunteer organizations, the most important of which are nongovernmental organizations (NGOs).^3^ NGOs are an essential element of modern governments’ management to improve social, political, and developmental activities. They play several important roles in the process of development in most countries^4^ and global health governance. NGO activities can be local, national, or international. NGOs have contributed to the development of communities around the world and are important partners of many governments, while simultaneously remaining independent from governments.^5^

The Islamic Republic of Iran is also 1 of the countries where COVID-19 has become widespread and many people have died of it (deaths: 58,336 as of February 6, 2021). In Iran, as in other countries, NGOs have contributed to the health system in disease control since the beginning of the outbreak. There are almost 3000 NGOs involved in controlling COVID-19 in Iran. The most important activities of NGOs in Iran included: providing education and training to people, controlling people’s stress and psychological pressures, providing and distributing health-care materials, disinfecting cities and villages, fundraising for the health system, patient screening, cooperation for food supply, preparing and distributing of essential materials and goods needed by the people, advocating and communicating with national and international officials as the role of NGOs in controlling COVID-19, and the formation of National Service Network (consisting of approximately 2000 specialized and nonspecialized NGOs to help control the pandemic).

There are several high impact recommendations for improving the ability of NGOs to assist health-care systems with COVID-19 and future pandemic response, including increasing financial and medical equipment support, improving training across all responding organizations for better alignment and integration, and publicly requesting support for health-care systems that are particularly fragile. Other actions that states should take include improving registration systems for NGOs, sharing communication systems with all responders for effective coordination, and increasing collaboration between all responders to enhance the effectiveness of health-care system response.
